# A meta-analysis of Prognostic factor of Pancreatic neuroendocrine neoplasms

**DOI:** 10.1038/s41598-018-24072-0

**Published:** 2018-05-08

**Authors:** Yong Gao, Hao Gao, Guangfu Wang, Lingdi Yin, Wenbin Xu, Yunpeng Peng, Junli Wu, Kuirong Jiang, Yi Miao

**Affiliations:** 1 Pancreas Center, The First Affiliated Hospital of Nanjing Medical University, Nanjing, 210029 People’s Republic of China; 2 Pancreas Institute of Nanjing Medical University, Nanjing, 210029 People’s Republic of China; 3 Department of General Surgery, The First Affiliated Hospital of Nanjing Medical University, Nanjing, 210029 People’s Republic of China

## Abstract

Pancreatic neuroendocrine neoplasms (pNENs) are a group of clinically rare and heterogeneous diseases of the pancreas. However, the prognostic factors for this disease in patients still remain controversial. The purpose of our study is to evaluate the predictive roles of those prognostic factors for pNENs. All related articles published until Sep 17, 2017 were identified via PubMed, EMBASE, Web of Science, Ovid and the Cochrane Library. Studies that examined the prognostic factors of pNENs were enrolled. 17 articles (2822 patients) were finally included in this study. The pooled data suggested that patients with positive surgical resection margin and lymph node, advanced G stage and TMN stage, organ metastasis, vascular invasion and the necrosis of specimens had a decreased overall survival for pNENs. Similarly, patients with functional tumors might have a poor prognosis. However, age, gender, surgical type and size of tumor could not be regarded as prognostic factors for pNENs. Our analytic data demonstrated that surgical resection margin, G stage, TMN stage, lymph node, metastasis, vascular invasion and the necrosis could be prognostic factors for pNENs. Our study may assist doctors to screen patients with different prognosis more efficiently during follow-up and select appropriate treatment measures.

## Introduction

Pancreatic neuroendocrine neoplasms (pNENs), derived from different neuroendocrine cells, are a clinically rare and heterogeneous disease of the pancreas^1,2^. Since Seale Harris *et al*. the first time describing endogenous insulinoma in 1924, more and more subtypes of pNENs have been reported such as glucagonoma, gastrinoma, VIPoma, Somatostatinoma and CCK-oma^3^.

While the percentage of PNENs in all pancreatic tumors is only 1% to 2%, the incidence has increased apparently in the past few years^4,5^. Meanwhile, the interest on pancreatic neuroendocrine neoplasms (pNENs) has grown^6^. However, we still lack understanding of this disease.

The prognosis of pNENs in the population varies. As reported in some studies^7–10^, a large number of factors including older age, large tumor size, positive resection margin, advanced G stage and TMN stage, vascular invasion, organ metastasis could indicate a poor prognosis of pNENs. In contrast, people with function tumors, low tumor marks might have a better outcome. Unfortunately, the results of these studies were controversial, and the sample size of them was relatively limited. Effective prognostic factors are in great need. Thus we performed this meta-analysis to further evaluate the predictive roles of these prognostic factors for pNENs.

## Result

### Study selection

**Figure 1:**
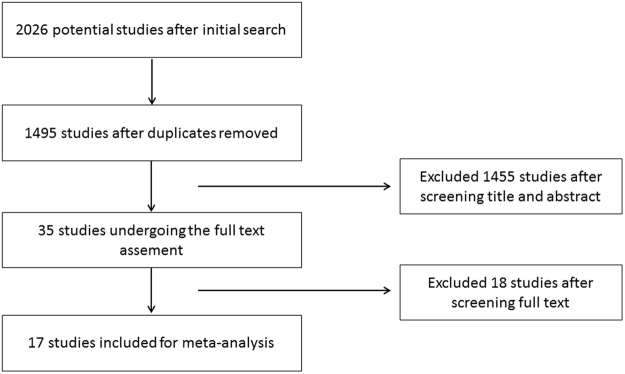
The identification and selection process for this meta-analysis.

The identification and selection process for this meta-analysis are illustrated in Fig. 1. A total of 2026 publications were identified in the initial literature search. Based on screening of titles or abstracts, 1455 records were excluded. 35 articles were left for the further full text assessment. According to the inclusion and exclusion criteria mentioned in materials and methods, 18 articles were excluded and 17 articles were finally included for this meta-analysis.

### Study characteristics

The main characteristics of these included articles were shown in Table 1. A total of 17 studies with 2822 patients (range from 19–1483 per study) were assessed in this meta-analysis^11–27^. Studies included were published from 2007 to 2017. Study period ranged from 1964 to 2015. Among them, 11 studies studied the patients from Asian countries, including Japan (2), South Korea (1), and China (8); 7 studies investigated the patients from European countries and North American countries, including America (3), Germany (2), Spain (1) and Norway (1). All of studies were retrospective studies. Moreover, the results of study quality assessment were also listed in Table 1.

**Table 1:** Main characteristics of included articles.

Author	Year	Country	Study period	Sample size	Study type	Quality assessment
Tao Ming *et al*.	2014	China	2007–2013	32	retrospective	7
Nils D. Arvold *et al*.	2011	USA	1983–2010	46	retrospective	6
Marcus Bahra *et al*.	2007	Germany	1989–2003	19	retrospective	6
Raziye Boyar Cetinkaya *et al*.	2014	Norway	1982–2010	114	retrospective	8
Cheng Yugang *et al*.	2016	China	2003–2015	100	retrospective	8
Javier A. Cienfuegos *et al*.	2016	Spain	1993–2015	79	retrospective	7
Resit Demir *et al*.	2011	Germany	1964–2006	82	retrospective	7
GaoChuntao *et al*.	2010	China	1980–2003	112	retrospective	8
GeWenhao *et al*.	2017	China	2007–2013	53	retrospective	7
T. R. Halfdanarson *et al*.	2008	USA	1973–2000	1483	retrospective	8
HanXu *et al*.	2017	China	2004–2013	104	retrospective	8
Satoshi Shiba *et al*.	2016	Japan	1991–2010	100	retrospective	8
Katsunobu Taki *et al*.	2017	Japan	2001–2014	83	retrospective	7
Joyce Wong *et al*.	2014	USA	1999–2012	131	retrospective	8
Yang M. *et al*.	2015	China	2002–2012	125	retrospective	8
YangMin *et al*.	2014	China	2000–2013	55	retrospective	6
ZhouBo *et al*.	2017	China	2002–2013	104	retrospective	8

### Prognostic factors for pancreatic neuroendocrine neoplasms

#### clinical feature prognostic factors

3 clinical feature prognostic factors were analyzed in this study, including gender, age, and function. All pooled data about these factors were shown in Table 2.

**Table 2:** Pooled data about clinical feature prognostic factors.

Factor	Number of articles	OR	95% CI	P	I^2^(%)
Gender	4	1.65	0.64–4.27	0.3	67
Age	4	1.03	1.00–1.05	0.03	45
Function	7	0.75	0.63–0.90	0.002	37

#### Gender

**Figure 2:**
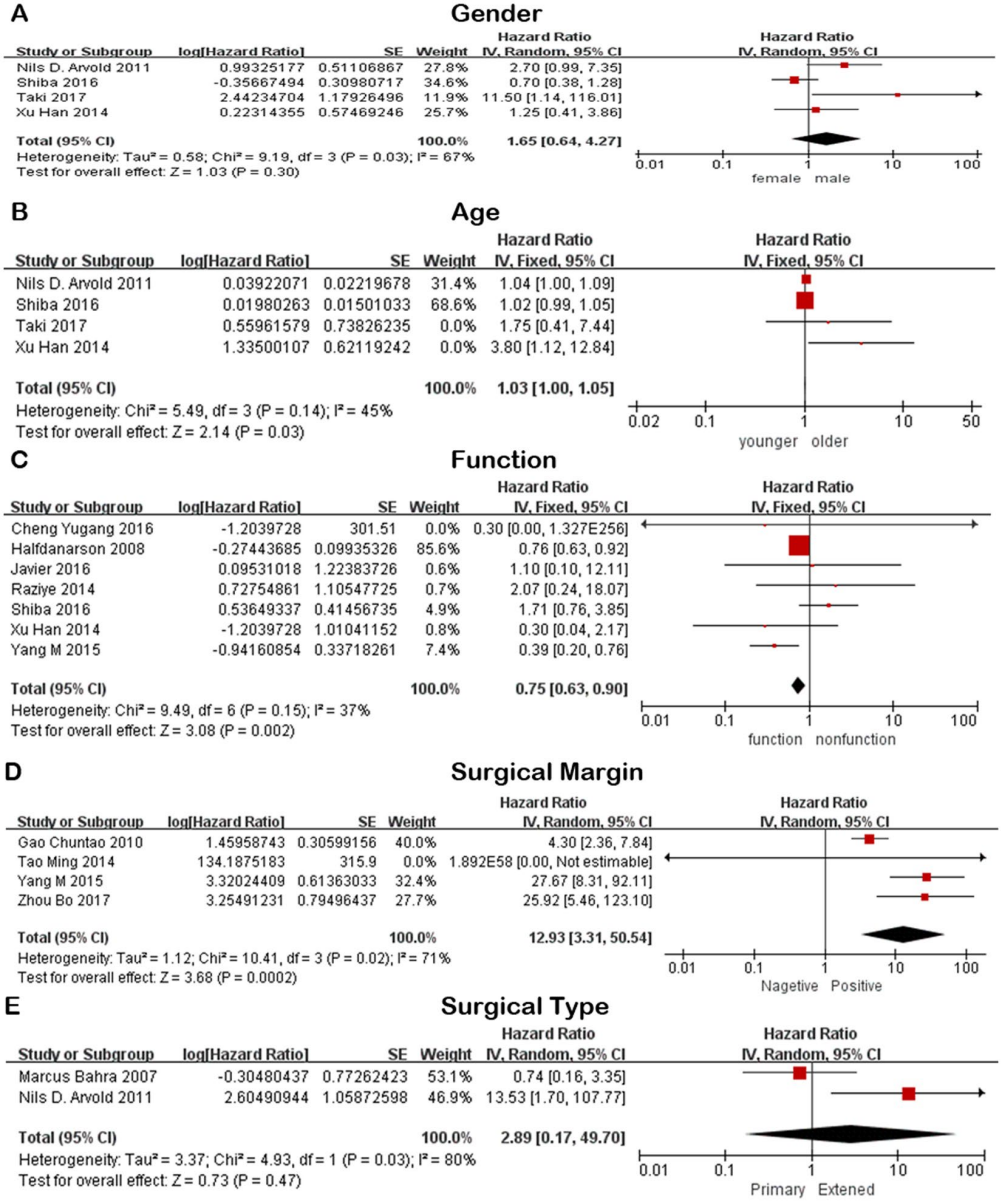
Forest plot of the association between pNENs and clinical feature and surgery prognostic factors. (**A**) The association between pNENs and gender. (**B**) The association between pNENs and age. (**C**) The association between pNENs and function. (**D**) The association between pNENs and surgical margin. (**E**) The association between pNENs and surgical type.

A total of 4 articles^13,17,18,21^ assessed the effect of gender on the prognosis of pNENs. 3 of them^13,17,18^ considered that there was no difference in prognosis between male and female patients and the pooled hazard ratio (HR) and 95% confidence interval (CI) supported this view. The rest one^21^ showed that male patients might have a poor prognosis of pNENs (Table 2, Fig. 2A). According to the subgroup analysis, equal survival tendency to males and females was only observed in results obtained from studies with larger sample size, and performed in Asia area.

#### Age

1 of 4 articles^13^ suggested that patients with older age had a worse outcome than the patient with younger age (Table 2, Fig. 2B). However, the combined data and all subgroup results all showed that there was no significant difference between young patients and old patients.

#### Function

Function was enrolled in 7 articles^11,13,14,16,18,22,27^. Although up to 5 of 7 articles^11,13,18,22,27^ found no statistically significant difference in the prognosis of functional and nonfunctional tumors, the combined result reported that functional tumors could improve the prognosis of pNENs (Table 2, Fig. 2C), as well as the subgroup results based on the articles enrolled more than or equal to 80 patients or published in western countries. However, articles published in Asia agreed with the previous point.

#### Surgery related prognostic factors

2 surgery related prognostic factors were investigated in our study. All synthetic data about these factors were shown in Table 3.

**Table 3:** Synthetic data about surgery related prognostic factors.

Factor	Number of articles	OR	95% CI	P	I^2^(%)
Surgical margin	4	12.93	3.31–50.54	0.0002	71
Surgical type	2	2.89	0.17–49.70	0.47	80

#### Surgical margin

1 of 4 article^12^ showed that there was no significant difference between patients with positive and negative surgical resection margin (Table 3, Fig. 2D). However, the combined result and subgroup result based on articles more than 80 patients all revealed that negative surgical margin was another positive prognostic factor for pNENs.

#### Surgical type

Nils D. Arvold *et al*.^17^ suggested that more extensive surgery was associated with decreased OS but the study^23^ carried by Marcus Bahra *et al*. showed inconsistent trend (Table 3, Fig. 2E).

#### Pathology related prognostic factor

7 pathology related prognostic factors were assessed in our study. All combined data about these factors were shown in the Table 4.

**Table 4:** Combined data about pathology related prognostic factors.

Factor	Number of articles	OR	95% CI	P	I^2^(%)
G stage	6	5.43	2.46–11.99	<0.0001	2
TMN stage	4	8.56	2.00–36.71	0.004	0
Lymph node	6	2.96	1.25–7.03	0.01	93
Vascular invasion	3	1.93	1.14–3.28	0.01	47
Organ metastasis	4	4.5	2.65–7.62	<0.0001	36
Tumor size	5	1.02	1.00–1.04	0.009	0
Necrosis	2	3.58	1.86–6.89	0.0001	0

#### G stage

**Figure 3:**
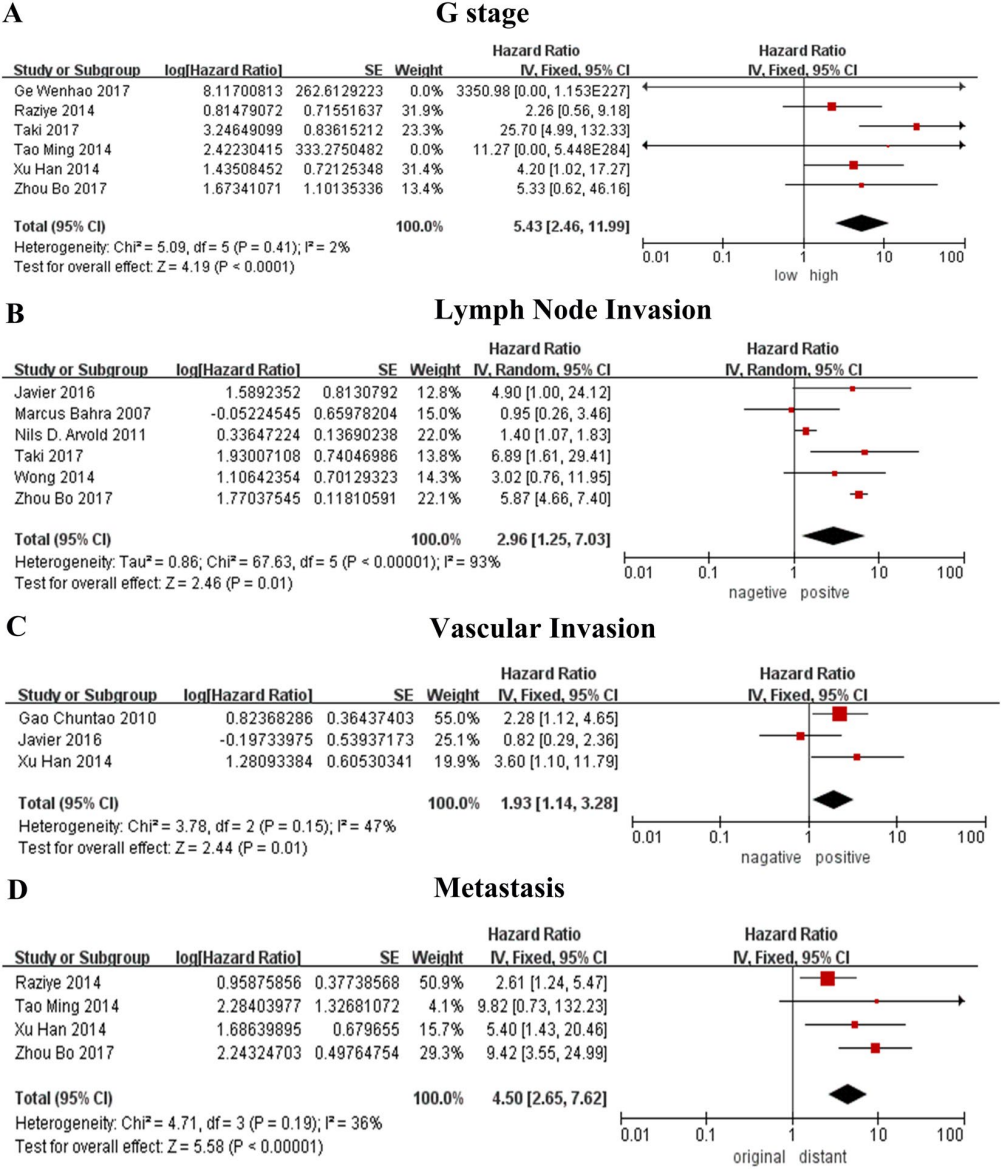
Forest plot of the association between pNENs and pathology related prognostic factor. (**A**) The association between pNENs and G stage. (**B**) The association between pNENs and lymph node invasion. (**C**) The association between pNENs and vascular invasion. (**D**) The association between pNENs and metastasis.

2 of 6 studies^13,21^ suggested that poor prognosis of pNENs was associated with advanced G stage, while 4 of 6 studies^12,20,26,27^ had no statistical difference between people with high or low G stage. Our pooled data showed that patients with advanced G stage were prone to suffer from pNENs (Table 4, Fig. 3A). Meanwhile, subgroup analysis suggested that G stage is a negative prognostic factor for pNENs according to the pooled data from articles published in Asian and articles with more than or equal to 80 patients

#### TMN stage

TMN stage was mentioned in 4 articles^18,22,24,25^. On the basis of current knowledge, people with advanced TMN stage are more likely to have a negative outcome. However, only 1 article^18^, along with combined data and subgroup analysis approved this standpoint. The rest articles showed no difference.

#### Lymph node

While 2 of 6 articles^19,23^ showed no difference in people with or without lymph node invasion, 4 articles^17,20–22^ reflected that people with lymph node invasion had worse prognosis than people without lymph node invasion. The combined data subordinate to the majority (Table 4, Fig. 3B). In the subgroup analysis, poor prognosis was associated with the people with lymph node invasion based on the articles with large samples and studies conducted in Asian or western countries.

#### Vascular invasion

2 articles^13,25^ with more than 80 patients supported that vascular invasion involvement is related to a poor outcome. In contrast, 1 article^22^ with small samples suggested that there is no difference in people with or without vascular invasion (Table 4, Fig. 3C).

#### Organ Metastasis

Present organ metastasis might indicate low overall survival of pNENs according to the data provided by 3 of 4 articles^13,20,27^ and the combined data (Table 4, Fig. 3D). All subgroup analysis also showed the identical results.

#### Tumor size

5 studies^17,19,21,24,26^ were mentioned in our study. 4^19,21,24,26^ of them showed no correlation with the size of tumor and prognosis of pNENs.1 of them suggested that people with tumor in large size might have a poor prognosis.

#### Necrosis

Study^17^ carried by Demir *et al*. showed that necrosis was a negative prognostic factor for pNENs. The other article^13^ written by Xu Han *et al*. hold a conservative attitude.

## Discussion

Pancreatic neuroendocrine tumors are rare and heterogeneous tumors with poorly defined natural history and uncertain biological behavior^28,29^. With advances in imaging techniques, pNETs are now being detected with increasing frequency in many regions of the world^30^. However, there is still no set of standard rules to determine the prognosis of patients. In this study, we reviewed several related prognostic factors for pNENs to further confirm their roles.

As far as we know, we, for the first time, assessed the significance of prognostic factors above for pNENs in a meta-analysis. Based on the combined HR and 95% CI, we believe that negative prognostic factors consists of positive surgical resection margin and lymph node, advanced G stage and TMN stage, present organ metastasis, vascular invasion and the necrosis of specimens. Thus, patients with aforementioned factors should gain more attention and be examined more frequently during the follow-up. However, the only one positive prognostic factor is function. Therefore, patients with non-functional tumors should also show more concern on themselves.

Furthermore, our study found that many other prognostic factors such as age, gender, surgical type and size of tumor do not play a decisive role in the process of pNENs. We might have a conservative view for these factors due to the lack of enrolled studies and the limited sample size. Therefore, more studies about some potential prognostic factors were greatly needed, especially studies with a large number of patients.

To reduce the effect of small samples and regional disparity on heterogeneity, we performed the subgroup analysis based on sample size and nationality of patients. For almost all of subgroup analysis, we found that there is a small change in the value of combined 95% CI based on the large samples and patients in Asia or western countries, but the relationship between 95% CI and 1 hasn’t changed. Therefore, there is no decisive effect on heterogeneity in the studies based on small sample and geographic distance. For function, the nationality of patients was identified as main factor resulting in heterogeneity. Of course, many other factors could also result in the heterogeneity, such as year of articles.

There are some limitations concerning this study. Firstly, some articles only provide Kaplan-Meier curve but not HR or follow-up data. During the process of computation, we may increase the deviation from the original data. In addition, many other factors might be responsible for the overall survival. However, due to the lack of sufficient articles or effective data, they were not assessed in our study. Moreover, the cutoff value to define high and low or positive and negative varies among some studies, which increased the difficulty with performing a pooled study. Last but not least, randomized controlled trials were in need to improve the reliability of reported data.

In conclusion, this meta-analysis indicates that positive surgical resection margin and lymph node, advance G stage and TMN stage, present organ metastasis, vascular invasion and the necrosis of specimens might be associated with a poor prognosis of pNENs.

These findings will provide important theoretical basis for improvement in clinical follow-up of patients with pNENs and may increase overall survival in a long term. However, due to the limitations mentioned above, further well-designed studies with larger sample size are required to confirm the predictive roles of those factors.

## Materials and Methods

### Literature search

Potential studies were selected by screening PubMed, EMBASE, Web of Science, Ovid, and The Cochrane Library dating up to September 2017. In Pubmed and Cochrane Library, the following keywords were applied in searching: (neuroendocrine tumors[Title/Abstract] OR neuroendocrine tumor[Title/Abstract] OR neuroendocrine neoplasms[Title/Abstract] OR neuroendocrine neoplasm[Title/Abstract]) AND (prognosis[Title/Abstract] OR prognostic factor[Title/Abstract]). In Web of Science and Ovid, we used the following search strings: (neuroendocrine tumors[Title]OR neuroendocrine tumor[Title] OR neuroendocrine neoplasms[Title] OR neuroendocrine neoplasm[Title]) AND (prognosis[Title] OR prognostic factor[Title]). In EMBASE, we searched for neuroendocrine neoplasms OR neuroendocrine neoplasm OR neuroendocrine tumor OR neuroendocrine tumors AND prognosis. The last search was performed on Sep 17, 2017. In addition, the search results were supplemented by examining references mentioned in the original articles.

### Inclusion and exclusion criteria

Studies were included if they met the following criteria:

- pancreatic neuroendocrine tumor was histopathologically diagnosed.
- Relevant risk estimated in terms of hazard ratio with 95% confidence interval, or KM curve was provided.

The following criteria were applied to exclude studies:

- no full text or available data
- non-English language
- conference abstract or review
- basic research or preclinical research.

### Study quality assessment

The quality of included studies was assessed by two independent reviewers according to the Newcastle-Ottawa Quality Assessment Scale for cohort studies (NOS) recommended in the Cochrane Handbook version 5.1.0^31^. NOS is comprised of three parameters (eight elements, nine stars total) for quality: selection (four elements, one star each), comparability (one element, up to two stars) and outcome (three elements, one star each). The high-quality choices for each element are marked with a star, and then the number of stars is counted to evaluate the quality of each study. Studies are regarded as high quality if they are awarded six stars or more^32^.

### Data extraction

Two independent researchers carefully reviewed each eligible article and extracted the data. Any controversial data were resolved by a third researcher. For each enrolled study, the basic information extracted was shown: author name, year of publication, country, study types, study period and sample size. Potential prognostic factors mentioned in more than or equal to two articles were recorded. Gender, age, function, surgical type and margin, G and TMN stage, lymph node and vascular invasion, organ metastasis, tumor size and necrosis were finally selected. Subsequently, the number of articles, Hazard ratio, 95% confidence interval (CI) were acquired for each prognostic factor. If the data could not be obtained directly, we extracted survival rates from Kaplan-Meier survival curve, imported the data into Engauge Digitizer 4.1 to and calculated relative values with the method mentioned in article published by Jayne F Tierney *et al*.^33^.

### Statistical analysis

Review Manager software (version 5.3; Cochrane Collaboration, Oxford, United Kingdom) was applied to perform this meta-analysis and provide related graphics. Combined hazard ratio is presented as forest plots. Subgroup analysis was performed stratifying on the sample size (>80 vs. <80) and study area(Asian area vs. Western area). Cochran’s Q test and Higgins’ I-squared test were used to test heterogeneity between studies. Heterogeneity would not be considered significant if the *P* -value for Cochran’s Q test was greater than or equal to 0.1. In the absence of statistically significant heterogeneity, a fixed effects model was used to combine the data. Otherwise, a random effects model was applied.

## Electronic supplementary material

Supplementary Table
