# Supplementary material for: A meta-analysis of Prognostic factor of Pancreatic neuroendocrine neoplasms
Source: Sci Rep. 2018 May 8;8:7271. doi: 10.1038/s41598-018-24072-0 (PMC5940798; doi:10.1038/s41598-018-24072-0)
Supplement: Supplementary file 1 — Supplementary Table [file 41598_2018_24072_MOESM1_ESM.pdf]

**A meta-analysis of Prognostic factor of Pancreatic neuroendocrine neoplasms**

Yong Gao[1, 2, 3, +], Hao Gao[1, 2, 3, +], Guangfu Wang[1, 2, 3, +], Lingdi Yin[1, 2, 3], Wenbin Xu[1, 2, 3], Yunpeng Peng[1, 2, 3], Junli Wu[1, 2, 3], Kuirong Jiang[1, 2, 3], Yi Miao[1, 2, 3, *]

[1]Pancreas Center, The First Affiliated Hospital of Nanjing Medical University, Nanjing, 210029, People's Republic of China.

[2]Pancreas Institute of Nanjing Medical University, Nanjing, 210029, People's Republic of China.

[3]Department of General Surgery, The First Affiliated Hospital of Nanjing Medical University, Nanjing, 210029, People's Republic of China.

[*] corresponding. miaoyi@njmu.edu.cn

[+]these authors contributed equally to this work

| Factor | Number of articles | OR | 95%CI | P | $I^2$(%) |
|---|---|---|---|---|---|
| Gender | 3 | 1.38 | 0.44-4.36 | 0.58 | 65 |
| Age | 3 | 1.59 | 0.69-3.69 | 0.28 | 60 |
| Function | 6 | 0.75 | 0.63-0.90 | 0.002 | 47 |
| Margin | 3 | 13.05 | 3.15-54.08 | 0.004 | 80 |
| G stage | 4 | 5.43 | 2.46-11.99 | <0.0001 | 41 |
| Lymph Node | 3 | 5.79 | 4.62-7.26 | <0.0001 | 60 |
| Vascular Invasion | 2 | 2.57 | 1.40-4.75 | 0.002 | 0 |
| Metastasis | 3 | 4.81 | 2.07-11.15 | 0.0003 | 54 |

Supplementary Table 1 shows the combined data based on the articles in large samples.

| Factor | Number of articles | OR | 95%CI | P | $I^2$(%) |
|---|---|---|---|---|---|
| Gender | 3 | 1.38 | 0.44-4.36 | 0.58 | 65 |
| Age | 3 | 1.59 | 0.69-3.69 | 0.28 | 60 |

| | | | | | |
|---|---|---|---|---|---|
| Function | 4 | 0.66 | 0.22-1.98 | 0.46 | 64 |
| G stage | 5 | 8.19 | 3.14-21.36 | <0.0001 | 0 |
| TMN stage | 3 | 8.56 | 2.00-36.71 | 0.004 | 14 |
| Lymph Node | 3 | 5.79 | 4.62-7.26 | <0.0001 | 0 |
| Metastasis | 3 | 7.91 | 3.73-16.81 | <0.0001 | 0 |
| Size | 4 | 0.22 | 0.03-1.42 | 0.11 | 0 |

Supplementary Table 2 shows the Pooled data based on the articles in Asian area.

| Factor | Number of articles | OR | 95%CI | P | $I^2$(%) |
|---|---|---|---|---|---|
| Function | 3 | 0.77 | 0.63-0.93 | 0.007 | 0 |
| Lymph Node | 3 | 1.51 | 0.86-2.68 | 0.15 | 26 |

Supplementary Table 3 show the Combined data based on the patients from western country.
